# Blood Flukes Exploit Peyer's Patch Lymphoid Tissue to Facilitate Transmission from the Mammalian Host

**DOI:** 10.1371/journal.ppat.1003063

**Published:** 2012-12-20

**Authors:** Joseph D. Turner, Priyanka Narang, Mark C. Coles, Adrian P. Mountford

**Affiliations:** Centre for Immunology and Infection, Department of Biology and Hull York Medical School, University of York, York, United Kingdom; University of Medicine & Denistry New Jersey, United States of America

## Abstract

Schistosomes are blood-dwelling parasitic helminths which produce eggs in order to facilitate transmission. Intestinal schistosomes lay eggs in the mesenteries, however, it is unclear how their eggs escape the vasculature to exit the host. Using a murine model of infection, we reveal that *Schistosoma mansoni* exploits Peyer's Patches (PP) gut lymphoid tissue as a preferential route of egress for their eggs. Egg deposition is favoured within PP as a result of their more abundant vasculature. Moreover, the presence of eggs causes significant vascular remodeling leading to an expanded venule network. Egg deposition results in a decrease in stromal integrity and lymphoid cellularity, including secretory IgA producing lymphocytes, and the focal recruitment of macrophages. In mice lacking PP, egg excretion is significantly impaired, leading to greater numbers of ova being entrapped in tissues and consequently, exacerbated morbidity. Thus, we demonstrate how schistosomes directly facilitate transmission from the host by targeting lymphoid tissue. For the host, PP-dependency of egg egress represents a trade-off, as limiting potentially life-threatening morbidity is balanced by loss of PP structure and perturbed PP IgA production.

## Author Summary

Schistosomes are parasitic helminths that parasitise >200 million people worldwide. Adult worm pairs of intestinal schistosomes lay their eggs in the mesenteric veins from which the eggs need to pass into the lumen prior to excretion and completion of their life cycle. However, it is not known how eggs transfer from the intestinal vasculature to reach the gut lumen. Here, we reveal using a mouse model of infection, that *Schistosoma mansoni* exploits Peyer's Patches (PP) lymphoid tissues in the wall of the small intestine as a preferential route of egg egress. The eggs cause vascular remodelling in the PP leading to an expanded venule network, reduced stromal integrity, and decreased lymphoid cellularity. Most significantly, in mice rendered deficient in PP, egg excretion is impaired (despite intact immune responses), leading to greater numbers of eggs entrapped in tissues, and consequently exacerbated host morbidity. Thus, we demonstrate how schistosomes directly facilitate transmission from the host by targeting lymphoid tissue. For the host, this represents a trade-off as limiting life-threatening morbidity is balanced by loss of PP structure and function. The requirement of PP for efficient schistosome egress may be a significant risk factor of developing severe disease within heavily infected human populations.

## Introduction

Schistosomes are large metazoan pathogens of humans that parasitise the blood of >200 million people worldwide [1]. Unlike the majority of infections caused by viruses, bacteria and protozoa, schistosome infections are chronic, with a fecund worm life span of more than 10 years, and re-infections are frequent in areas of ongoing exposure [1]. Transmission of schistosomes from the mammalian host occurs via the excretion of 200–300 eggs per day, however approximately 90% of *Schistosoma mansoni* and *S. japonicum* infections yield a mild ‘intestinal’ disease. More severe ‘hepatosplenic’ and ‘chronic fibrotic’ schistosomiasis occurs in 10% of infections due to the accumulation of backwashed eggs into the liver, causing ∼280,000 deaths, annually [1], [2].

Adult *S. mansoni* worm pairs lay their eggs in the mesenteric veins from which they need to extravasate, traverse intestinal tissue and pass into the lumen prior to excretion. This process is initially dependent upon the host's immune responses as egg escape requires intact CD4^+^ T-helper 2 (Th2) lymphocyte responses [3], [4] and the subsequent ‘alternative activation’ of intestinal macrophages [5]. Egg excretion is tightly co-ordinated to coincide with full development of miracidia within mature ova [6], and certain molecules secreted by mature eggs [7] actively drive the development of polarized Th2-granulomatous responses [8], [9]. Thus, the mechanism of egg transmission represents an evolutionary adaptation of the parasite to exploit the host's adaptive immune system. However, as re-infections accumulate, and chronicity of disease progresses, anti-egg Th2 responses become impaired due to host immuno-regulatory processes and anergy [10]–[12]. Paradoxically, despite diminished host Th2 immunological reactivity at the chronic phase and much reduced granulomatous reactions around freshly laid ova, egg transmission is not significantly impeded [13], [14]. Thus, how the parasite has adapted to sustain egg excretion during a modulated Th2 response characteristic of chronic human infections is not understood.

Peyer's Patches (PP) are an important component of mammalian Gut-Associated Lymphoid Tissues (GALT) and are important sites for homeostatic and pathogenic secretory immunoglobulin A (sIgA) responses in the gut [15], [16]. In humans, PP numbers fluctuate with age, peaking at an average of 239 in puberty before declining to between 100–150 in adulthood [17]. Several intracellular, microbial pathogens (e.g. *Salmonella*, reoviruses and prion neurological agents), hijack PP tissues to facilitate dissemination from the gut lumen to internal organ sites [15]. Since PP aggregate in the ileum [18], which is a preferential site for egg deposition during human *S. mansoni* infection [19], we hypothesised that intestinal schistosomes could also exploit PP to promote transit of eggs from intestinal tissue to complete their life cycle.

## Results/Discussion

### Schistosomes deposit eggs abundantly in PP-associated vasculature and induce vascular remodeling

**Figure 1:**
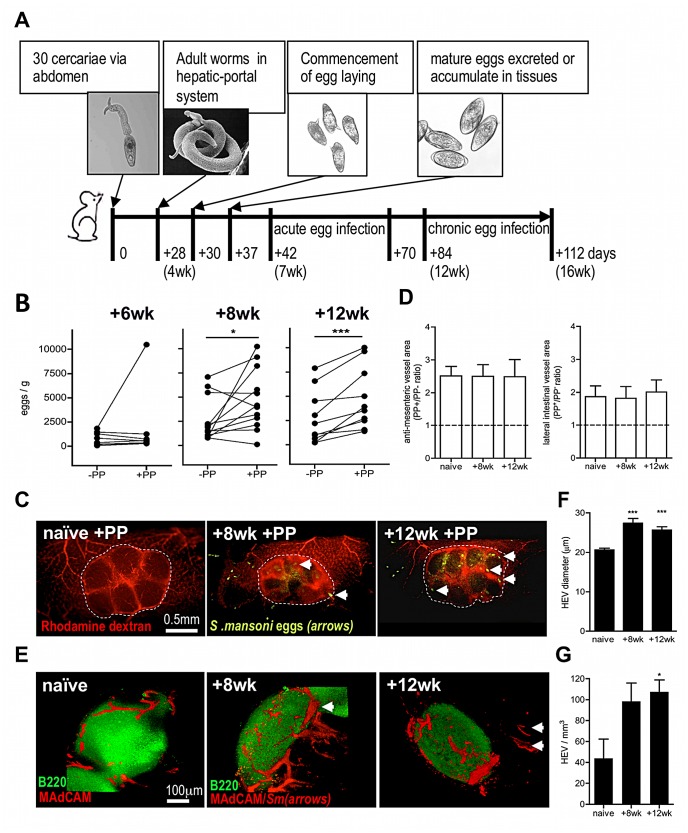
Egg deposition is more abundant within PP-associated vasculature. (A) Outline of experimental *S. mansoni* infection. (B) Numbers of eggs/g intestinal segments containing PP (+PP), or adjacent segments lacking PP (−PP) at 6, 8, and 12 weeks post-infection (pi). Data for −PP and +PP gut samples from the same mouse shown by connecting lines, n = 10–12, *P<0.05 or ***P<0.001, paired T test. (C) Angiograms of +PP vasculature (rhodamine dextran; red) in naïve mice, or 8 and 12 weeks pi. Dotted line demarks PP; arrows indicate schistosome eggs (auto-fluorescence). (D) Ratio of vascular area in PP+/PP− gut (imaged at the anti-mesenteric serosal face or 90° lateral to the anti-mesenteric face) based on angiograms in naïve mice or 8 and 12 weeks pi; min 3 PP/mouse. (E) 3D confocal images of PP stained for B cell follicles (green B220+) and HEV (red MAdCAM+); Arrows = schistosome eggs (auto-fluorescence). (F & G) Quantification of HEV from 3D rendered confocal images of gut sections stained for MAdCAM+ (red), ***P<0.001, *P<0.05 cf. naïve, 1-way ANOVA with Tukey's post-hoc. Data is mean (+SEM), n = 3–4 mice/group and is representative of 2–3 independent experiments.

Using a murine *S. mansoni* infection model (outlined in Figure 1A), we assessed whether egg deposition varies between adjacent sections of small intestine containing a PP (+PP) or not (−PP), by dissecting equivalent sized sections of each after establishment of a patent infection. The number of eggs increased >2-fold by 8 weeks, and >3-fold by 12 weeks, in +PP compared with −PP gut tissue (Figure 1B). As the greater burden of eggs within +PP gut could be due to an increase in the abundance of vasculature supplying GALT, we performed fluorescent angiograms on naïve or infected mice (Figure 1C) which demonstrated that the total vascular area of +PP compared with −PP gut sections was ∼2.4-fold more abundant at the anti-mesenteric surface, and ∼1.8 more abundant at the lateral surface, irrespective of infection status (Figure 1D). However, 3 dimensional (3D) imaging of MAdCAM^+^ (mucosal cell-adhesion molecule-1) High Endothelial Venules (HEV) within +PP tissue after infection demonstrated major remodeling of the venous vascular network (Figure 1E), evident as ∼1.3-fold increases in HEV diameter at 8 and 12 weeks (Figure 1F, Movies S1, S2, S3), and increases in their overall number (Figure 1G). Thus, we conclude that since egg-deposition is more abundant in +PP gut tissue, this reflects increased vascularity available to migratory adult worms in gut tissue with PP (*cf.* without PP) and increased access of an expanded venule network in PP induced after the onset of egg production.

### PP-localised egg deposition leads to damage and loss of cellularity

**Figure 2:**
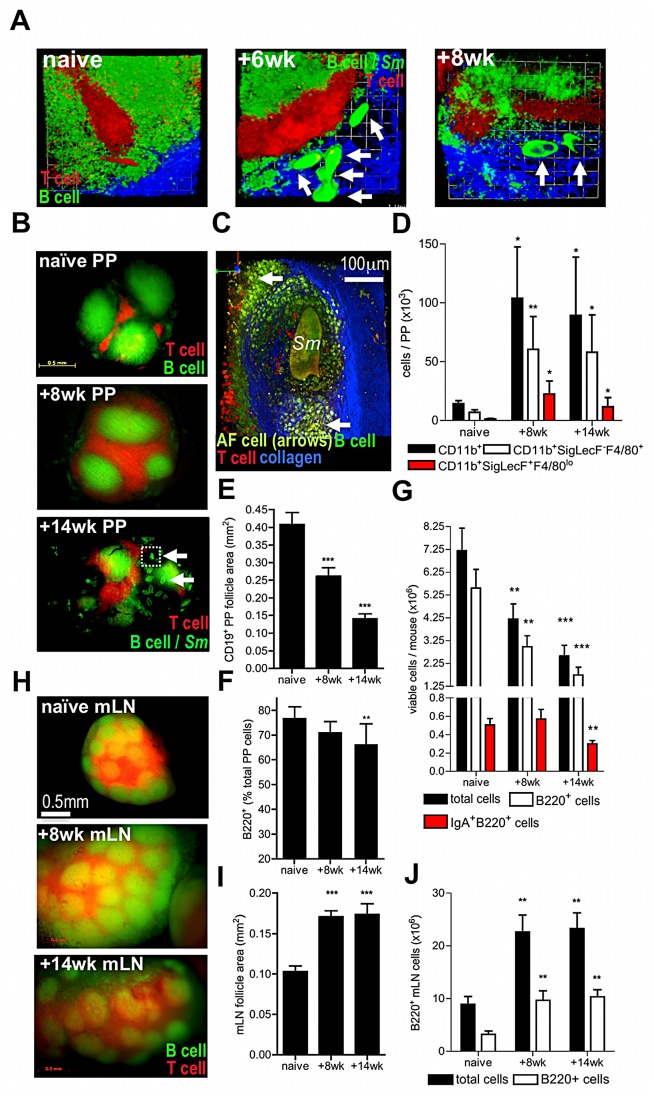
Egg deposition within PP disrupts lymphoid microarchitecture and causes loss of cellularity. (A) DsRed and eYFP expression demarcate B cell follicles and inter-follicular T cell zones of PP. Images are 3D rendered multi-photon confocal display of excised PP tissue derived from VaDsRed/CD19+ eYFP double fluorescent reporter mice (red = CD3+ cells; green = CD19+ cells; blue = second-harmonic generation by collagen fibres). Auto-fluorescent egg (arrows) proximal to PP at 6 and 8 weeks pi. All data are representative of two independent experiments, n = 3 mice. (B) Stereo fluorescent stereomicroscope images of PP in naïve, and infected CD3+/CD19+ reporter mice. Auto-fluorescent eggs (arrows) co-localise to T and B cell zones of PP at 14 weeks (lower panel). (C) 3D multiphoton confocal image of PP egg-granuloma (enlargement of section inset shown in B as dotted line); Sm = autofluorescent egg; arrows = cellular infiltrate; blue = second-harmonic generation by collagen fibres. (D) Numbers of CD11b+ cells, CD11b+SigLecF-F4/80+ macrophages, and CD11b+SigLecF+F4/80lo eosinophils in PP cell suspensions enumerated by flow cytometry in naïve mice, or after infection. (E) CD19+ PP follicle area in double reporter mice (min 3 PP/mouse). (F) Total viable PP, B220+ and IgA+B220+ B cell yields/mouse and (G) proportion of B220+ cells of total PP. (H) Stereo fluorescent stereomicroscope images of mLN *in situ* from naïve and infected CD3+ (red)/CD19+ (green) reporter mice. (I) mLN follicle area, (J) total viable cell and B220+ B cell yields in the mLN of naïve and infected CD3+T cell/CD19+B cell double reporter mice. All data; *P<0.05, **P<0.01, ***P<0.001, cf. naïve, 1-way ANOVA with Tukey's post-hoc. Bars are means (+SEM), n = 3–5, representative of 2–3 independent experiments.

PP were examined in infected hCD2-VaDsRed/CD19-eYFP double fluorescent reporter mice, where >90% of CD3^+^ T cells express DsRed and >80% of CD19^+^ B cells express eYFP, in order to assess cellular and pathological changes in PP after deposition of schistosome eggs (Figure 2A–C). At 6 weeks, recently laid immature eggs were deposited high up within intestinal venules and were evident in chains leading to and surrounding PP (Figure 2A). By 8 weeks, mature egg stages were detected adjacent to the PP T cell zone (Figure 2A); however by 14 weeks, eggs were present in both the T cell zones and B cell follicles (Figure 2B). In addition, auto-fluorescent cells coalesced around mature eggs and collagen remodeling was apparent, both indicative of granuloma formation within PP (Figure 2C, Movie S4). Analysis of the infiltrating cells revealed a 10-fold increase in the abundance of CD11b^+^ myeloid cells, consisting mainly of F4/80^+^SigLecF^−^ macrophages and F4/80^lo^SigLecF^+^ eosinophils at both 8 and 14 weeks (Figure 2D, Figure S1). Furthermore, there was a progressive diminution in PP area and B cell follicle size (Figure 2E) illustrating that egg deposition adversely affects PP homeostasis. At 14 weeks, there was a ∼2-fold reduction in total PP cellularity, a ∼3-fold reduction in B220^+^B cell number, including IgA-secreting B220^+^ cells (Figure 2F), and a 10.6% mean reduction in B cell proportion compared with naïve mice (Figure 2G). However, loss of GALT integrity was limited to PP (and ‘PP-like’ caecal tissues; data not shown), as mesenteric lymph nodes (mLN) in the same mice had intact microarchitecture and increased total cellularity (Figure 2H–J), as well as a significant abundance of F4/80^lo^SigLecF^+^ myeloid cells (data not shown).

**Figure 3:**
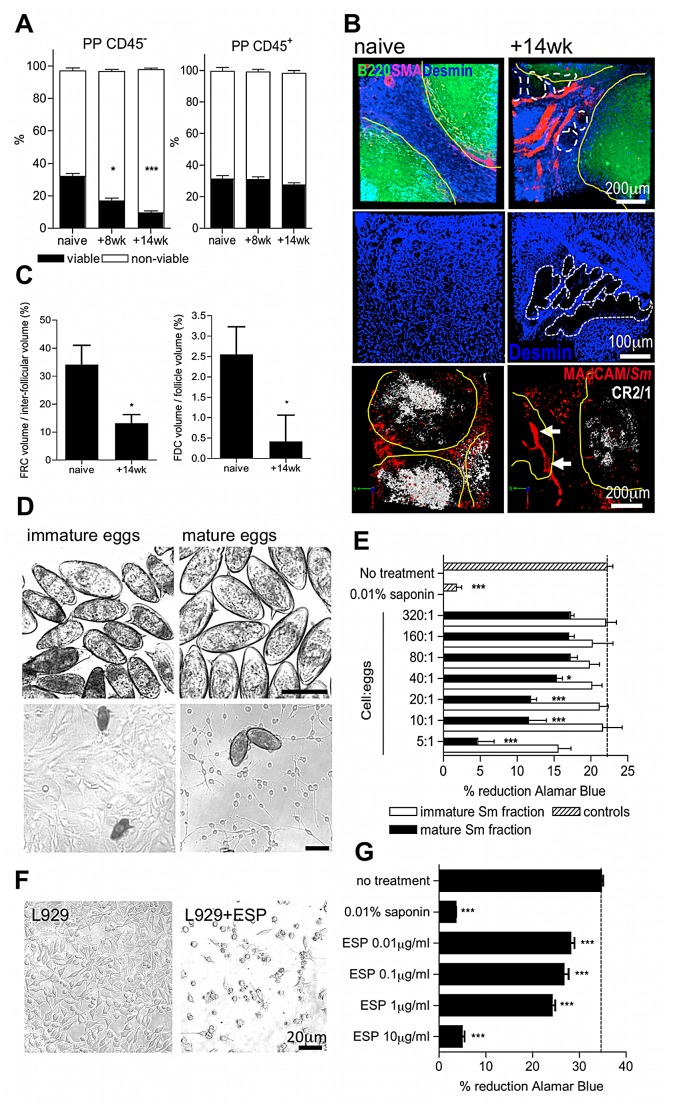
Egg deposition and their secretions elicit damage to PP stroma. (A) Viable (annexinV−/propidium iodide−) CD45− but not CD45+ cells decline in PP cell suspensions after infection; *P<0.05, ***P<0.001, cf. naïve, 1-way ANOVA with Tukey's post-hoc. (B) Upper panels: 3D confocal imaging of PP showing B220+ cell follicles (green, yellow outline), desmin+/smooth muscle actin− fibroblast-like reticular cells (FRC, blue) and desmin+/smooth muscle actin+ blood vessel basement membranes (pink). Dashed lines demarcate void space in FRC tissue. Middle panels: high magnification of desmin+ FRC within PP interfollicular region. Dashed lines demarcate void space in FRC tissue. Lower panels: 3D confocal imaging of PP showing B220+ cell follicles (yellow outline), CR2/1^hi^ follicular dendritic cells (FDC, white) and MAdCAM+ high endothelial venules (red). Intravascular *S. mansoni* eggs (red autofluoresence) are indicated by arrows. (C) FRC+ and FDC+ tissues as a proportion of inter-follicular volume, or follicular volume: min 3 PP/mouse, *P<0.05, cf. naïve, unpaired T-test. Representative of 2–3 independent experiments, n = 4–5. (D) Phase-contrast images of immature or mature eggs co-cultured with OP9 bone marrow fibroblast stroma (bars = 100 µm). (E) Metabolic activity of fibroblasts 72 h after co-culture as determined by Alamar Blue. (F) Phase contrast images of L929 fibroblasts 72 h post culture in the presence, or absence, of 10 µg/ml secretions from mature eggs (ESP). (G) Metabolic activity determined by Alamar Blue reduction of L929 fibroblasts co-cultured for 72 hr with different concentrations of ESP; untreated control = dashed line. *P<0.05, ***P<0.001, cf. naïve or untreated control, 1-way ANOVA with Tukey's post-hoc. Data are means (+SEM), n = 3–5, representative of 3 independent experiments.

Because non-haematopoietic stromal cells (i.e. CD45^−^) are vital in the organisation of B and T cell areas in secondary lymphoid tissues [20], [21], we reasoned that egg transit might damage the stromal compartments of PP. Indeed, there was a progressive deterioration in the viability of CD45^−^ stromal cells in the PP following the onset of egg deposition which was not reflected by changes in the viability of CD45^+^ hematopoietic cells (Figure 3A, Figure S2), or CD45^−^ stromal cells in the mLN (data not shown). Immunolabelling PP sections for fibroblast-like reticular cells (FRC; desmin^+^ smooth muscle actin^−^) revealed a loss of T cell stroma by 14 weeks after infection (Figure 3B–C); void space indicative of egg-induced damage and mechanical displacement was apparent in FRC tissue adjacent to the vasculature (shown as dashed line upper and middle panel Figure 3B). Examination of follicular dendritic cell (FDC; complement receptor 2/1^hi^B220^−^) stromal compartments within PP follicles also revealed a substantial loss following chronic egg deposition (lower panel Figures 3B & 3C). Damage to resident PP stromal compartments could be caused by release of cytotoxic products from recruited granuloma cells in contact with eggs. Alternatively, or in addition, it could be due to molecules released by maturing eggs (egg secretory products; ESP) which include a T2-class ribonuclease with demonstrated hepatotoxicity [22] and proteins with putative protease-like function [7]. To test the hypothesis that eggs are an agent of stromal cytotoxicity, immature eggs were separated from mature eggs by density centrifugation, before co-culturing with OP9 fibroblasts (Figure 3D). Mature, but not immature eggs, inhibited the normal proliferation of these cells (Figure 3E). Moreover, secretions collected from mature eggs (ESP) also inhibited the growth of L929 fibroblast cultures (Figures 3F & G). We therefore conclude that the deleterious effects on GALT following schistosome infection are directly driven by localised egg deposition, and propose that egg secretions mediate damage to PP stroma leading to loss of lymphoid cellularity.

### Lack of PP results in reduced egg transmission and increased morbidity

**Figure 4:**
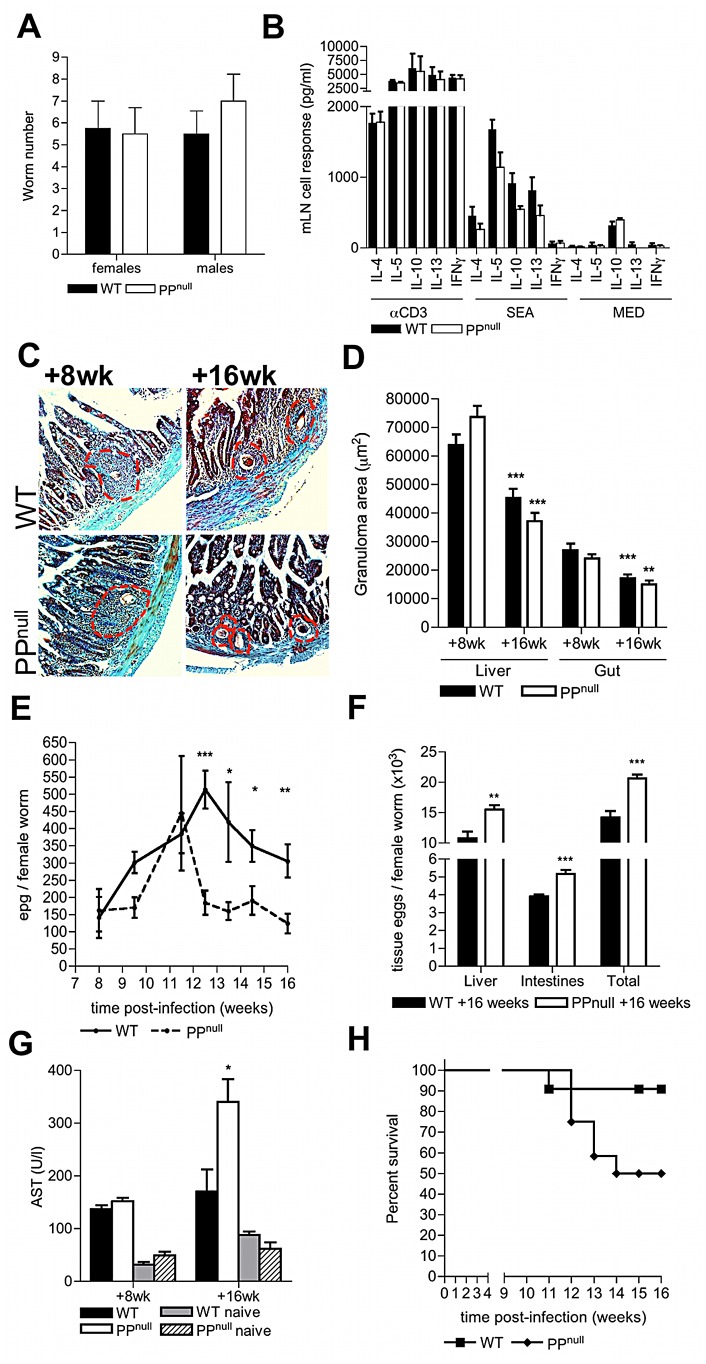
PP are required to sustain optimum egg transmission and limit host morbidity. (A) Quantitative analysis of adult schistosome worm recoveries following perfusion of the hepatic portal system in WT or PP^null^ mice. (B) Cytokine secretion by mLN cells from WT and PP^null^ mice 8 weeks post-infection following in vitro stimulation with anti-CD3 mAb, or soluble egg antigen. (C) Masson's Trichrome stained histological sections of duodenum (blue = collagen) at post-infection in WT or PP^null^ mice (red dotted lines = extent of egg granulomas). (D) Reduction in granuloma areas at 16 cf. 8 weeks; min 4 granulomas/mouse, **P<0.01, ***P<0.001, unpaired T test: nd = no difference between WT and PP^null^. (E) Numbers of faecal eggs from WT and PP^null^ mice 8–16 weeks pi, and (F) tissue eggs in the small intestine and liver at week 16; both normalised to female worm burden. (G) Levels of AST enzyme in the serum of naïve or infected mice. (H) Incidence of morbidity of ‘moderate severity’; P = 0.0507, survival analysis. Statistical analysis is for PP^null^ versus WT mice, *P<0.05, **P<0.01, ***P<0.001, unpaired T test of log10-transformed data (E) or raw data (D, F, G & H). Data is mean+SEM of two independent experiments, (A, B & D; n = 4), or combined data of two independent experiments (E–H; n = 7–13).

As PP-associated vasculature is clearly a favoured site for schistosome egg deposition resulting in damage to the PP stroma/microarchitecture, we reasoned that schistosomes might exploit these conditions to aid transit of their eggs from the intestinal environment. To test this, mice with a specific deficiency in PP (PP^null^) were created by *in utero* antibody blockade of IL-7 receptor-dependent PP organogenesis; this protocol prevents the formation of PP but not other lymphoid tissue such as the mLN [21] (Figure S3). The development of schistosome worms was not affected in PP^null^ mice demonstrating that transient IL-7R blockade had no influence on the maturation, or number of adult worms (Figure 4A, & Figure S3). Moreover, after infection, PP^null^ mice had intact type-2 inflammatory mLN responses to soluble egg antigen (Figure 4B), mounted comparable intestinal and hepatic granulomatous reactions (Figures 4C & D), and exhibited similar down-regulation of granuloma size by week 16 (Figures 4C & D). Together these data demonstrate that a). mLN-specific immune responses are unaltered following *in utero* IL-7R blockade, b). PP are not necessary for host anti-egg granulomatous responses, and c). PP are not required to mediate down-regulation of granulomatous inflammation. In contrast, egg excretion from the intestines was significantly impaired in PP^null^ mice from week 12 onwards (Figure 4E). As a consequence of impaired egress, significantly more tissue eggs were detected in the intestines and livers of PP^null^ mice at 16 weeks (Figure 4F). Moreover, elevated serum levels of the enzyme AST were also detected at 16 weeks in PP^null^ mice, indicative of increased hepatic dysfunction (Figure 4G). This enhanced egg-induced pathology culminated in increased overt morbidity of PP^null^ animals from >12 weeks (Figure 4H).

In conclusion, our data reveals that schistosome blood flukes exploit PP to aid the escape of the egg stage to the environment. To our knowledge, this is the first example of a macroparasite using lymphoid tissue to facilitate transmission, or of a pathogen transmitting from the blood to the environment via PP. Whilst schistosome eggs actively induce type-2 inflammation and alternative activation of macrophages in the gut via their secretions to initiate egg egress [8], [9], [23], escape via PP represents a unique process which becomes important for the maintenance of egg transmission during chronic infection. Considering the presence of immature eggs in intestinal venules as the ‘calling-cards’ of visitation by migratory worm pairs (or partially detached female worms), we define that PP are preferential sites of egg deposition by schistosomes as chronicity of infection progresses due to their greater abundance of vasculature as shown in the cartoon (Figure S4). Also we speculate that the remodeling of inflamed PP HEV further aids temporary accommodation of egg-laying female worms and their ova high up the PP –associated vasculature during progression of infection. Using the analogy of a sink filling with water at a rate faster than it can drain, PP^+^ gut vasculatures represent inherently larger sinks with greater total capacity for egg deposits, reducing the rate of ‘spill-over’ into hepatic tissues. Damage to PP stroma and declining PP cellularity occurs as a direct result of egg deposition. Loss of stromal cell viability in addition to mechanical displacement of stroma by egg deposition and granuloma formation is a probable factor in the overall loss of PP lymphoid cellularity because these tissues are fundamental as conduits for the trafficking of lymphocytes into secondary lymphoid tissue [24], [25] and their retention in defined T and B cell niches [20], [21]. As we found no evidence of reduced viability of the PP lymphoid cell compartment following chronic egg deposition, we propose cytotoxic effects of egg secretions mainly exert an effect on slow growing, resident stroma immediately adjacent to egg deposits rather than rapidly proliferating PP lymphoid cells and recruited granuloma cells. This does not rule out the possibility that inflammatory cells immediately adjacent to eggs may be adversely affected by secretions, rendering them dysfunctional in terms of anti-egg defences. The net effect of these pathological alterations is to provide a reduced tissue mass for eggs to traverse preceding extravasation. Also, we show that following egg deposition, PP act as a focus for recruitment of macrophages known to be critical in facilitating egg transit [5]. Further, granulomatous foci surrounding eggs deposited in PP are more florid than in the ileum and remain so even upon down-regulation in chronic disease [26] (our unpublished observations). Granuloma size has been shown to positively correlate with egg excretion rate in *S. mansoni* infection [27]. We speculate that in addition to increased access to vasculature for egg-laying worms, PP become favourable ‘portals’ for the transit of eggs which become important to maintain optimum egg egress though out chronic infection.

We suggest this mechanism is relevant to human intestinal schistosomiasis as we define two outcomes of egg transit through PP to be a). reduced host morbidity, and b). loss of PP-B cell homeostasis, including total IgA producing cells. The ramifications of loss of PP-IgA production are not known but may potentially impact on the ability of the chronically infected host to mount effective mucosal antibody responses to gut pathogens.

Our data suggests that heterogeneity in PP number in *S. mansoni*-exposed human populations is a possible risk factor for the development of severe hepatic morbidity in the chronically infected. Apart from *S. mansoni*, other schistosome species (*S. japonicum, S. intercalatum, S. mekongi*) also use the mesenteric/gut route to further their onward transmission and conceivably also utilize PP during chronic infections. Whilst the urinary schistosome, *S. haematobium* utilizes a distinct anatomical location of the bladder venous plexus to disseminate from the host, this does not rule out the possibility that abundant bladder mucosal-associated lymphoid tissues may be important in facilitating transmission of this parasite.

## Materials and Methods

### Ethics statement

All experiments were carried out in accordance with UK Animal's Scientific Procedures Act 1986 and with the approval of The University of York Ethics Committee.

### Experimental schistosome infections

C57BL/6 (B.6) and hCD2-VaDsRed/CD19-eYFP B.6 mice (dual T cell/B cell reporter mice; generated from crossing hCD2-VaDsRed mice (24), CD19cre (25) and Rosa26eYFP reporter mice) were maintained within the University of York under specific pathogen-free conditions. PP^null^ mice were the progeny of normal female B.6 mice given 0.5 mg intraperitoneal injections of protein-G affinity purified anti-IL-7R (hybridoma clone A7R34, ATCC) delivered to pregnant mice at days E14.5 and E16.5.

Female mice (8–10 weeks old) were infected percutaneously via the abdomen with 30 *S. mansoni* cercariae, and infections allowed to mature. Sampling of mice occurred between 8 to 16 weeks after infection. From +8 weeks, mice were assessed daily for loss of condition. Mice were euthanized if one of the following criteria was observed, defining suffering of ‘moderate severity’ as outlined in the Animal Scientific Procedures Project Licence: Weight loss >19% of body weight, measured against age matched controls; Staring coat – marked piloerection; Subdued – animal shows subdued behavior patterns, even when provoked; Hunched intermittently; Pallor, of eyes, nose, ears, and foot pads; Altered respiration – temporary or intermittent abnormal breathing pattern; persistent (>96 h) diarrhoea; bloody diarrhoea (>48 h).

### Parasitology

Egg burdens in weighed portions of liver, or washed intestinal tissues, were enumerated following overnight digestion of tissues in 4% KOH. Eggs in weighed faecal material (150–300 mg) from individual mice were enumerated following dispersion in PBS, filtration with several washes through 100 µm pore mesh, and concentration by centrifugation. Under terminal anaesthesia, adult schistosomes were recovered and quantified by perfusion of the hepatic-portal system with heparinised saline.

### Live *S. mansoni* egg isolation and culture

Hepatic *S. mansoni* eggs were isolated from +7 week infected NMRI outbred mice percutaneously exposed to 200 cercariae. Minced livers were washed 3x in pre-warmed Dulbecco's (D)PBS followed by overnight collagenase D digestion (1 mg/ml) in DPBS at 37°C. Digested livers were layered on top of 40% Percoll (GE Healthcare)/0.9% saline solution in DPBS and centrifuged at 300g for 15 minutes, room temperature with no brake. *S. mansoni* eggs were collected from the bottom of the Percoll gradient washed 3x in pre-warmed RPMI and layered onto 60% Percoll/0.9% saline solution in RPMI. Following centrifugation at 300*g*, 15 minutes, no brake, immature *S. mansoni* eggs were removed from the interface and mature eggs were isolated from the bottom of the column. Eggs were washed a total of 6x to remove residual Percoll. Immature egg enrichments typically contained 10–20% mature stages whereas mature fractions contained less than 1% immature eggs, as judged by microscopic examination of size and shape. Egg secreted products (ESP) of mature egg cultures were generated as described [7] and checked for purity by 1D-gel electrophoresis. Hatch assays of post-culture eggs confirmed that typically >80% of cultured eggs contained viable miracidia.

### Angiography and stereo microscopy

Mice were injected intravenously with 500 µg 10 kDa dextran labelled with rhodamine (Molecular Probes). After 5 minutes, mice were killed and the small intestines washed in ice cold PBS. Small intestines were then cut into three equal lengths, mounted onto chilled glass pipettes and imaged using a fluorescent stereo microscope with mercury light source and GFP and DsRed filter sets (Luminar, Zeiss). Images were collected with AxioVision Software (Zeiss). For angiograms, each PP-containing mounted gut section was imaged both at the anti-mesenteric face of the gut (containing the PP) and subsequently at a 90° rotation to capture the lateral face of gut containing PP. Angiograms were analysed using Volocity 5.5 (Perkin Elmer) with region of interest (ROI) and intensity threshold measurement tools. Minimum and maximum thresholds of rhodamine fluorescence were adjusted manually for each field of interest to distinguish signal from diffuse background autofluoresence. Caecal and mLN secondary lymphoid tissue were visualised following excision using fluorescent stereo microscopy and regions of fluorescence (indicating red T cell zones and green B cell zones) captured as described above.

### Immunostaining and confocal microscopy

Washed small intestinal tissues were fixed in 4% formal-saline, embedded in agarose and sectioned using a vibrotome (Leica) set at 150 µm intervals. Sections were blocked overnight in PBS containing 0.15% Triton X100 and 5% normal goat serum (Sigma) and then labelled overnight with combinations of the following monoclonal antibodies: rat anti-mouse MAdCAM-1 AlexaFluor488 (MECA367; BioLegend), rat anti-mouse CR2/1 Pacific Blue (eBio4E3; eBioscience), rat anti-mouse B220 AlexaFluor647 (RA3-6B2; eBioscience), or rat anti-mouse smooth muscle actin Cy3 (Sigma). Desmin was stained using rabbit anti-mouse desmin affinity-purified antibody (AbCam) followed by rat anti-rabbit AlexaFluor647 secondary antibody (Invitrogen). Sections were serially dehydrated with 25%, 50%, 75% and 100% methanol before being mounted within 10 mm aluminium depression slides. Reduction of tissue optical density was performed with a 1∶1 ratio of benzyl-alcohol/benzyl benzoate (BABB, Sigma). Fluorescence was captured using a 510 NLO Laser-Scanning Microscope (Zeiss). Baseline laser scanning settings were undertaken on isotype controls; resultant negative control images contained negligible fluorescent signal. Three-dimensional projections of PP tissues were rendered from z stacks using Volocity 5.5 software. Morphometric quantification of HEV numbers and vessel calibre were analysed using ROI and line tools within Volocity 5.5. Stromal volumes were performed using ROI and threshold intensity measurement tools within Volocity 5.5. Minimum and maximum threshold intensities were defined using naïve control PP tissue samples stained simultaneously as PP infected tissue samples. Threshold intensity settings were thus identical between all tissue samples quantified for the purposes of accurate statistical comparison.

For multiphoton imaging, small intestinal segments containing PP derived from naïve or infected hCD2-VaDsRed/CD19 eYFP double fluorescent reporter mice were mounted within 10 mm depression slides, and PP imaged from the serosal surface using a 510 NLO laser-scanning microscope (LSM, Zeiss) with multi-photon laser (Coherent) tuned to 872 nm. 3D projections of PP tissues were rendered from z stacks using Volocity 5.5 software.

### Histology

Small intestinal tissues were fixed in 4% formaldehyde and embedded in wax. Transverse cross-sections (5 µm) were stained with H&E, or Masson's Trichrome (Department of Veterinary Pathology, University of Liverpool). Digital photomicrographs of granuloma areas were analysed using Image J software. Pixel counting of collagen-specific staining was undertaken utilizing MatLab Software.

### Secondary lymphoid tissue cell isolations

Small intestines were flushed with ice cold PBS, mounted onto chilled Pasteur pipettes and PP excised from surrounding small intestinal tissue using iris scissors with the aid of a dissecting microscope. Single cell suspensions were prepared by mincing tissues before incubating in the presence of 1 mg/ml Collagenase D (Roche) for 30 minutes, 37°C, prior to passing through 70 µm cell strainers (BD Biosciences). Cell pellets were washed 3x in ice cold PBS, followed by centrifugation (300*g*, 5 minutes, 4°C). Spleen cell preparations were depleted of red blood cells by ammonium chloride lysis (ACK buffer, Pierce). Resultant single cell suspensions were enumerated by haemocytometer counting with trypan blue discrimination of non-viable cells.

### Flow cytometry

Cell suspensions were washed in ice-cold FACS buffer (PBS containing 0.5% BSA and 2 mM EDTA). Fc-receptor mediated antibody binding was blocked with anti-CD16/CD32 (eBioscience) at 0.5 µg/1×10^6^ cells, then aliquots of 0.25–0.5×10^6^ cells were labelled with combinations of fluorophore-conjugated rat anti-mouse CD11b (M1/70), F4/80 (BM8), CD45 (30-F11), B220 (RA3-6B2), (all eBioscience) and SigLecF (E50-2440; BD Bioscience) for 30 minutes in 100 µl FACS buffer on ice. Cells were washed in 1 ml FACS buffer and pelleted by centrifugation (300*g*, 5 minutes, 4°C) Apoptotic and necrotic cells were labelled by dual anti-annexin V FITC antibody/propidium iodide staining following manufacturer's instructions (Cell viability kit; BD Biosciences). Cells were analysed using a Cyan flow cytometer with Summit software (Beckman Coulter).

### mLN cell recall assays

Isolated mLN cells were seeded into 96-well plates (0.2×10^6^/well), in RPMI L-glutamine medium supplemented with 10% FCS, 50 µg/ml penicillin/streptomycin and stimulated with plate-bound anti-CD3 mAb (1 µg; Becton Dickinson), or SEA (50 µg/ml) as described (14). Cells were cultured for 72 h, 37°C, 5% CO2 and supernatants retained for cytokine analysis. ELISAs were used to quantify IL-4, IL-5 and IFNg (14), while IL-10 and IL-13 were measured by Cytoset (Invitrogen) or DuoSet (R&D Systems) kits respectively

### Murine fibroblast assays

Murine L929 fibroblasts (ECACC) or OP9GFP bone marrow fibroblasts were cultured in complete DMEM (2 mM L-glutamine, 10% FCS, 50 µg/ml penicillin/streptomycin). Cells were seeded into 96-well plates (1×10^3^/well) in 50 µl medium and allowed to adhere to wells by culture at 37°C, 5% CO_2_ for 2 h. S. mansoni egg fractions, ESP, 0.01% saponin (positive control), or medium (negative control), were added at indicated concentrations in an additional pre-warmed 50 µl volume. Fibroblasts were cultured for 72 h, after which, cellular metabolic activity was measured by Alamar Blue reduction (Serotec), following manufacturer's instructions.

### Liver function assay

Blood was collected into heparinised tubes by tail tip bleeds at indicated time points and plasma fractions removed after centrifugation (300*g*, 5 minutes, 4°C). Activity of circulating liver enzyme aspartate transaminase (AST), in 10 µl plasma was determined by enzymatic kinetic assay (Sigma) following manufacturer's instructions and measuring change in optical density at 340 nm every 60 seconds for 30 minutes (PolarStar Optima, BMG). AST (Sigma) was used as a positive control. The linear phase of spectrophotometric rate was determined and average rate change per minute calculated. From this, the concentration of AST in circulation was calculated as detailed in the manufacturer's instructions.

## Supporting Information

Figure S1 Gating strategy for determining the phenotype of myeloid infiltrating cells within PP cell suspensions prepared at given time points. Grey filled histograms are the isotype control, the hatched line histograms are for cells labelled with mAb against CD11b.
(TIF)

Figure S2 Gating scheme to determine viable, early apoptotic (annexin V+/propidium iodide−) and late apoptotic/necrotic (propidium iodide+) CD45+ and CD45− cells within PP, or mLN cell suspensions. Grey filled histograms are the isotype control; the red line histogram is for cells labelled with mAb against CD45.
(TIF)

Figure S3 A) PP ablation via timed *in utero* blockade of IL-7Rα. B) absence of PP in small intestines of progeny of anti-IL-7Rα treated pregnant female mice C) normal development of *S. mansoni* in PP^null^ mice.
(TIF)

Figure S4 Proposed model of the mechanism of egg-escape via PP in chronic schistosome infection.
(TIF)

Movie S1 3D confocal rendered image of a PP follicle and associated HEV network in a naive mouse. Green = B220+ B cells, red = MAdCAM+ HEV.
(MOV)

Movie S2 3D confocal rendered image of a PP follicle and associated HEV network 8 weeks following infection. Green = B220+ B cells, red = MAdCAM+ HEV. Also shown are intravascular eggs (red autofluoresence) within HEV.
(MOV)

Movie S3 3D confocal rendered images of a PP follicle and associated HEV network12 weeks following infection. Green = B220+ B cells, red = MAdCAM+ HEV.
(MOV)

Movie S4 3D multiphoton confocal rendered image of a *S. mansoni* egg granuloma localised in a PP follicle 14 weeks pi of hCD2+-VaDsRed/CD19+ eYFP double fluorescent reporter mouse, Green = CD19+ B cells, red = CD2+ T cells, light green/yellow autofluoresence = *S. mansoni* egg and infiltrating cells, blue autofluoresence = collagen second harmonic generation.
(MOV)
