# Supplementary figures and images for: Blood Flukes Exploit Peyer's Patch Lymphoid Tissue to Facilitate Transmission from the Mammalian Host
Source: PLoS Pathog. 2012 Dec 20;8(12):e1003063. doi: 10.1371/journal.ppat.1003063 (PMC3534376; doi:10.1371/journal.ppat.1003063)

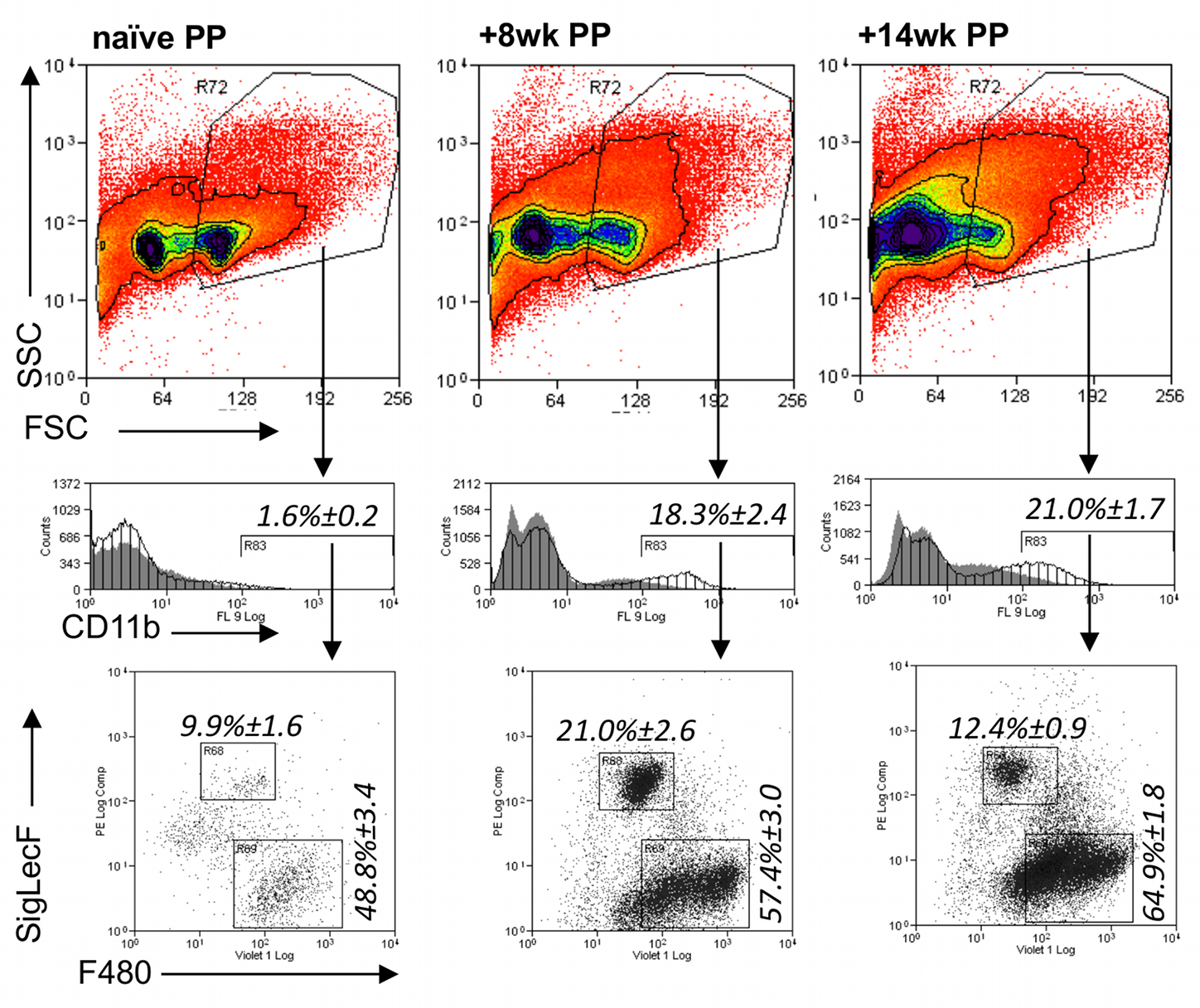

Supplement: Figure S1 — Gating strategy for determining the phenotype of myeloid infiltrating cells within PP cell suspensions prepared at given time points. Grey filled histograms are the isotype control, the hatched line histograms are for cells labelled with mAb against CD11b. (TIF) [file ppat.1003063.s001.tif]

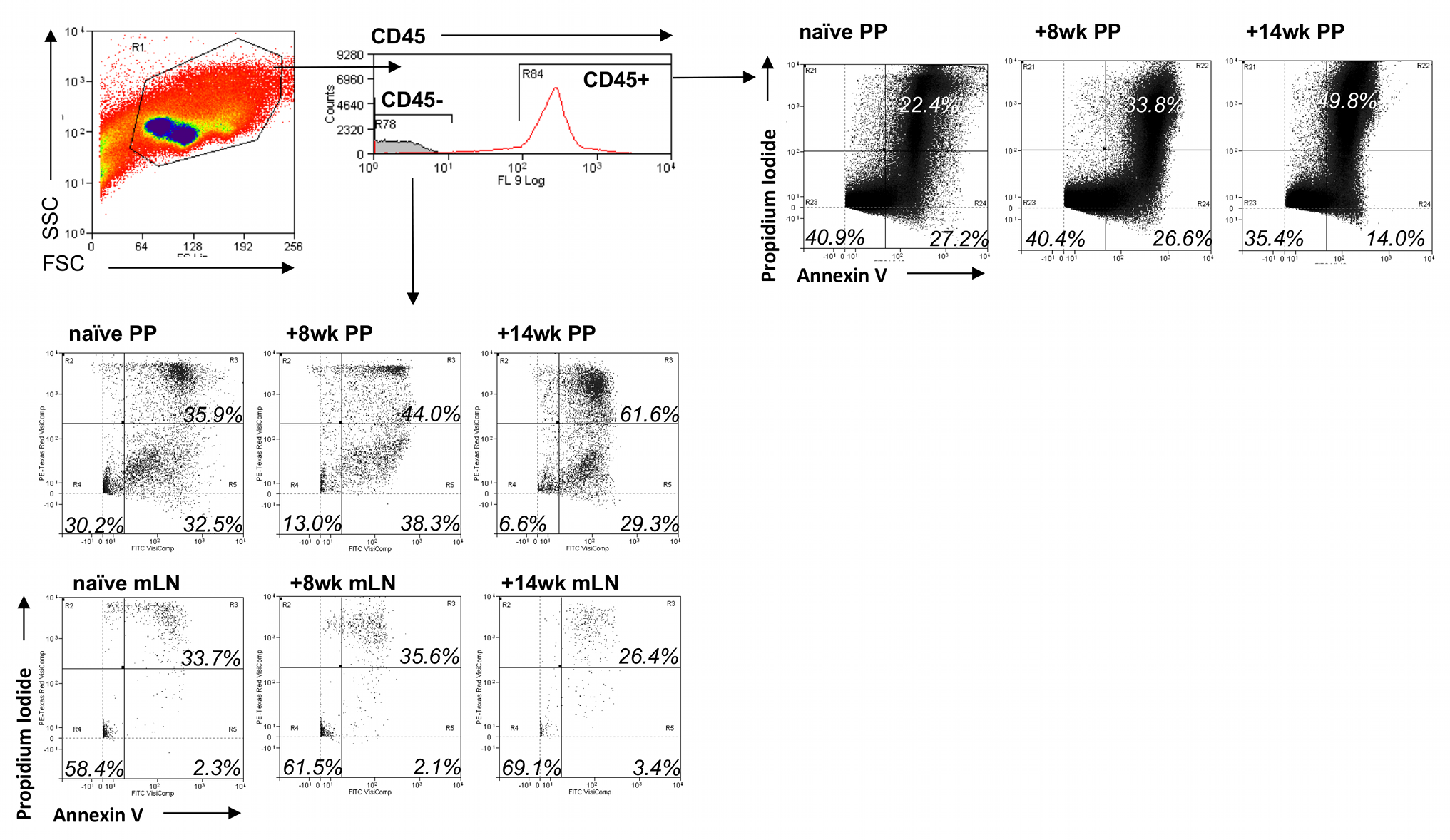

Supplement: Figure S2 — Gating scheme to determine viable, early apoptotic (annexin V+/propidium iodide−) and late apoptotic/necrotic (propidium iodide+) CD45+ and CD45− cells within PP, or mLN cell suspensions. Grey filled histograms are the isotype control; the red line histogram is for cells labelled with mAb against CD45. (TIF) [file ppat.1003063.s002.tif]

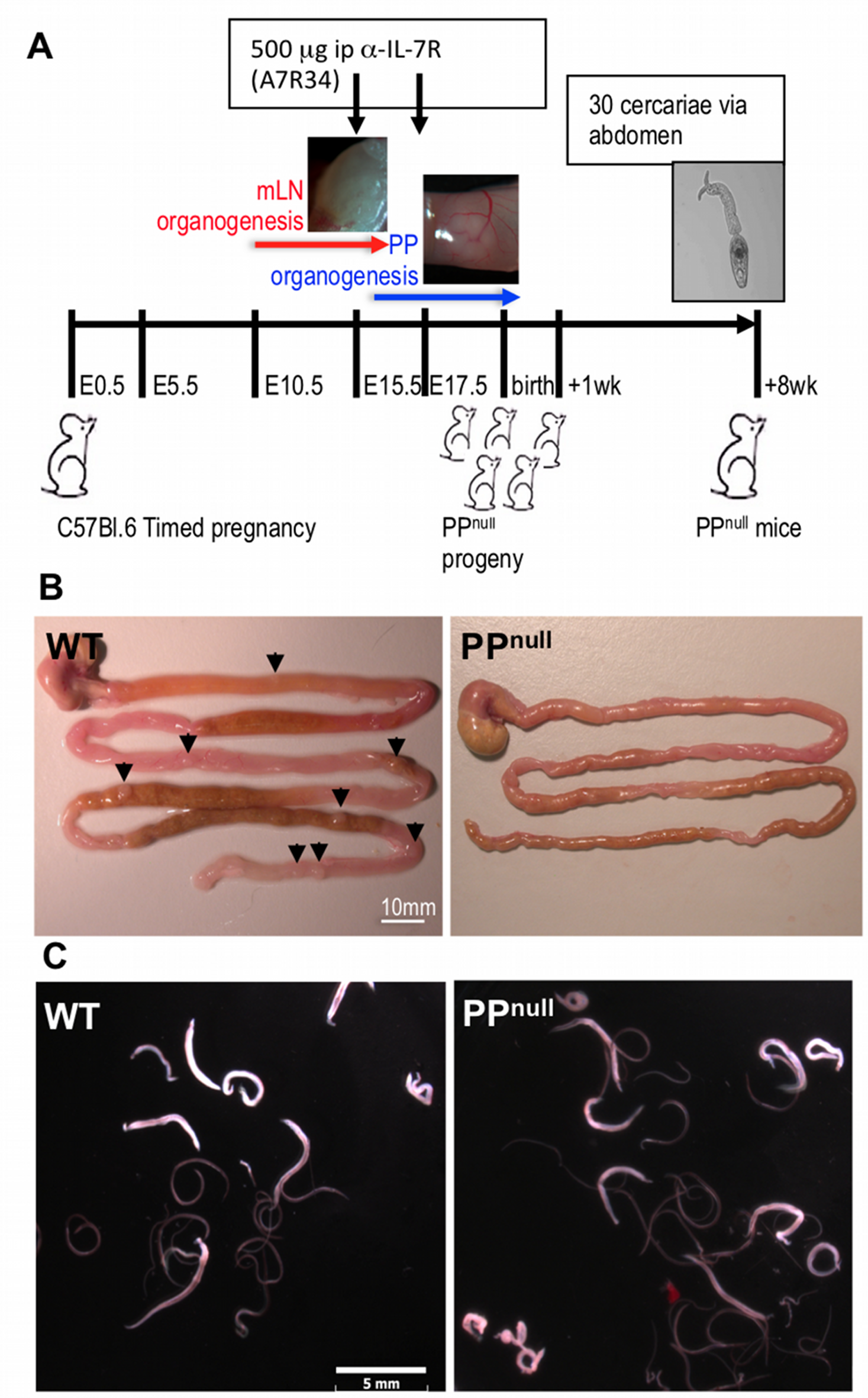

Supplement: Figure S3 — A) PP ablation via timed in utero blockade of IL-7Rα. B) absence of PP in small intestines of progeny of anti-IL-7Rα treated pregnant female mice C) normal development of S. mansoni in PPnull mice. (TIF) [file ppat.1003063.s003.tif]

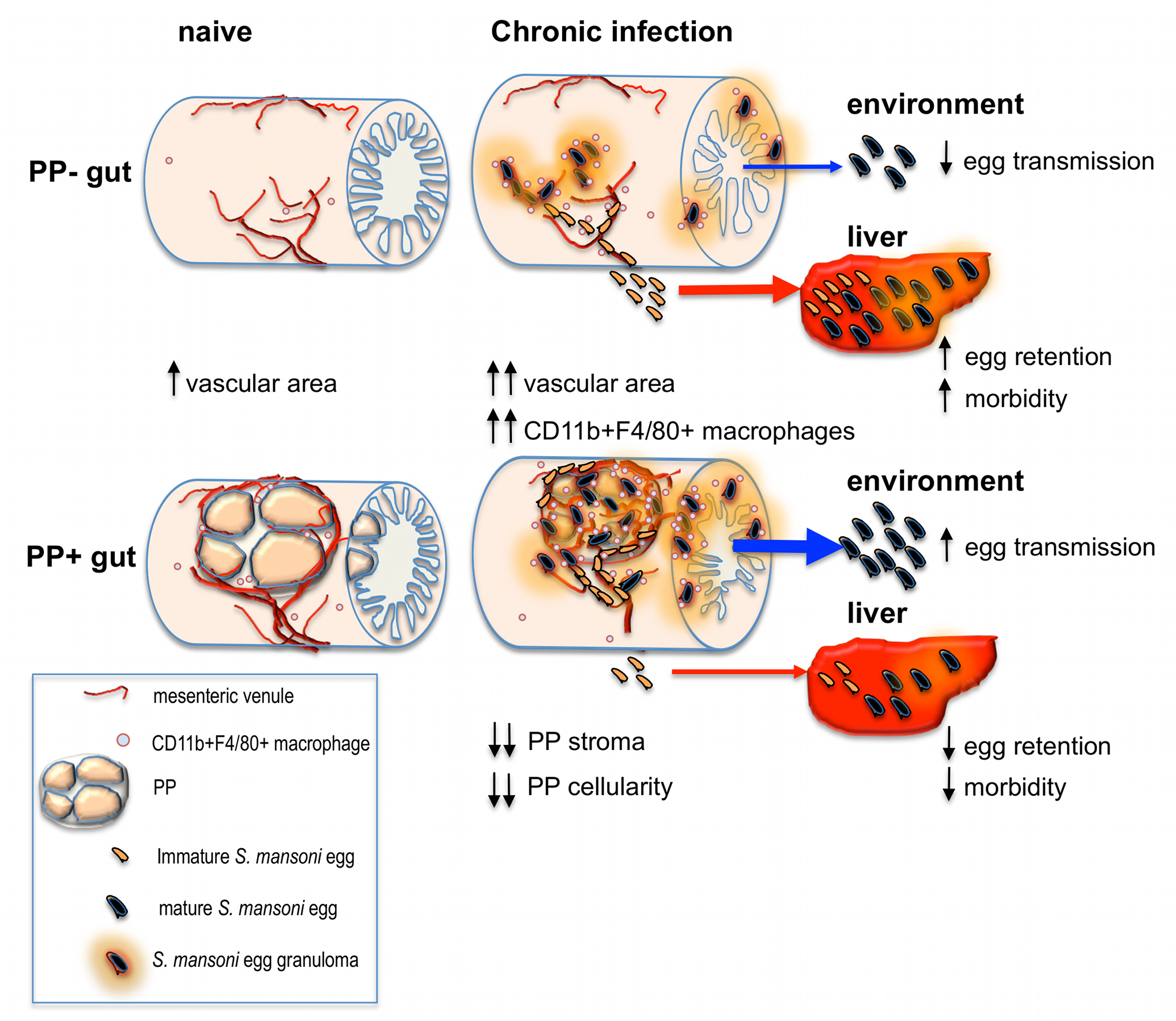

Supplement: Figure S4 — Proposed model of the mechanism of egg-escape via PP in chronic schistosome infection. (TIF) [file ppat.1003063.s004.tif]
